# Developmental Coordination Disorder, An Umbrella Term for Motor Impairments in Children: Nature and Co-Morbid Disorders

**DOI:** 10.3389/fpsyg.2016.00502

**Published:** 2016-04-15

**Authors:** Laurence Vaivre-Douret, Christophe Lalanne, Bernard Golse

**Affiliations:** ^1^Faculty of Medicine, University of Paris Descartes, Sorbonne Paris CitéParis, France; ^2^Institut National de la Santé Et de la Recherche Médicale UMR 1018 and CESP, University of Paris Sud-Paris Saclay, UVSQ and Paris Descartes, Sorbonne Paris CitéParis, France; ^3^Department of Child Psychiatry, AP-HP Necker-Enfants Malades University HospitalParis, France; ^4^Department of Pediatrics, Child Development, Cochin-Port Royal University Hospitals of Paris Center, Assistance Publique-Hôpitaux de ParisParis, France; ^5^Necker-Enfants Malades Hospital, University Hospitalo-Institut ImagineParis, France; ^6^Patient-Centered Outcomes Research, EA 7334 (REMES), University of Paris Diderot, Sorbonne Paris CitéParis, France

**Keywords:** developmental coordination disorder, neuro-developmental assessment, neuropsychological assessment, minor neurological dysfunctions, neurological soft signs, motor impairment, co-morbidity, brain MRI

## Abstract

**Background:** Developmental Coordination Disorder (DCD) defines a heterogeneous class of children exhibiting marked impairment in motor coordination as a general group of deficits in fine and gross motricity (subtype mixed group) common to all research studies, and with a variety of other motor disorders that have been little investigated. No consensus about symptoms and etiology has been established.

**Methods:** Data from 58 children aged 6 to 13 years with DCD were collected on DSM-IV criteria, similar to DSM-5 criteria. They had no other medical condition and inclusion criteria were strict (born full-term, no medication, no occupational/physical therapy). Multivariate statistical methods were used to evidence relevant interactions between discriminant features in a general DCD subtype group and to highlight specific co-morbidities. The study examined age-calibrated standardized scores from completed assessments of psychological, neuropsychological, and neuropsychomotor functions, and more specifically the presence of minor neurological dysfunctions (MND) including neurological soft signs (NSS), without evidence of focal neurological brain involvement. These were not considered in most previous studies.

**Results:** Findings show the salient DCD markers for the mixed subtype (imitation of gestures, digital perception, digital praxia, manual dexterity, upper, and lower limb coordination), vs. surprising co-morbidities, with 33% of MND with mild spasticity from phasic stretch reflex (PSR), not associated with the above impairments but rather with sitting tone (*p* = 0.004) and dysdiadochokinesia (*p* = 0.011). PSR was not specific to a DCD subtype but was related to increased impairment of coordination between upper and lower limbs and manual dexterity. Our results highlight the major contribution of an extensive neuro-developmental assessment (mental and physical).

**Discussion:** The present study provides important new evidence in favor of a complete physical neuropsychomotor assessment, including neuromuscular tone examination, using appropriate standardized neurodevelopmental tools (common tasks across ages with age-related normative data) in order to distinguish motor impairments gathered under the umbrella term of developmental coordination disorders (subcortical vs. cortical). Mild spasticity in the gastrocnemius muscles, such as phasic stretch reflex (PSR), suggests disturbances of the motor pathway, increasing impairment of gross and fine motricity. These findings contribute to understanding the nature of motor disorders in DCD by taking account of possible co-morbidities (corticospinal tract disturbances) to improve diagnosis and adapt treatment programmes in clinical practice.

## Introduction

According to current DSM criteria in Diagnostic and Statistical Manual of Mental Disorders (American Psychiatric Association, 2013) a diagnosis of DCD can be given to children who firstly exhibit marked impairment in the development of motor skills or motor coordination in comparison to peer groups (e.g., catching an object, using scissors or cutlery, handwriting, riding a bike, or participating in sports), although no cut-off exists (criterion A) and secondly, an interference with activities of daily living and impact on academic performance, prevocational and vocational activities, leisure, and play (criterion B). The onset of symptoms occurs in the early developmental period (criterion C). The motor skill deficits are not better explained by intellectual disability (intellectual developmental disorder) or visual impairment and are not attributable to a neurological condition affecting movement (e.g., cerebral palsy, muscular dystrophy, degenerative disorder) (criterion D).

DCD is defined as a failure to have ever acquired the ability to perform age-appropriate complex motor actions that is not explained by inadequate practice or demonstration.

There have been numerous attempts in the literature to define subtypes of DCD (Dewey and Kaplan, 1994; Hoare, 1994; Miyahara, 1994; Wright and Sugden, 1996; Macnab et al., 2001; Green et al., 2008; Vaivre-Douret et al., 2011a). The only common features between all these profiles are difficulties in sensorimotor processes reflected by performance scores for global and fine motricity, classified in a general DCD group. Usually, such measures are based on standardized motor performance scores, such as the Movement Assessment Battery for Children (M-ABC, Henderson and Sugden, 1992) or the Bruininks Oseretsky test of Motor Proficiency (BOTMP, Bruininks, 1978), and they are summarized as average profiles of performance for the same total score. It should be noted (Lyytinen and Ahonen, 1988; Vaivre-Douret et al., 2011a) that cluster research in these studies did not use the same complementary measures in addition to motor skill assessment (e.g., perceptual measures, as in the cluster studies by Hoare (1994), Macnab et al. (2001), Vaivre-Douret et al. (2011a) and/or transitive gestures in the cluster study by Dewey and Kaplan (1994) and Vaivre-Douret et al. (2011a). So it is difficult to compare the different cluster studies and DCD subtypes they propose. Furthermore, these studies did not look at co-morbidity phenomena or neurological soft signs (NSS) in the measures used for cluster analysis to understand the nature of the deficits.

Recent studies (Vaivre-Douret et al., 2011a,b; Lalanne et al., 2012) however provide a better understanding of the diagnostic criteria for DCD and its etiology for the identification of DCD subtypes using clinical and statistical approaches. Extensive assessments were conducted in these studies. Two pure subtypes of DCD/dyspraxia were distinguished: ideomotor (IM) and visuo-spatial/ constructional (VSC), and a mixed subgroup (MX) comparable to the general DCD group found in subtyping research, sharing common impairments (IM and VSC and global deficit in motricity) but with additional co-morbidities. Children suffering from ideomotor DCD appeared to form a rare group with marked impairments in digital perception, imitation of gestures, and digital praxis. The VSC subgroup was characterized by impairments in visual motor integration and visual spatial motor structuring tasks, and Lego blocks. The MX subgroup showed specific impairments in motor coordination of the lower and upper limbs, and poor manual dexterity.

Several major studies in the literature on DCD using cluster analysis to define subtypes did not use a complete standardized developmental examination including clinical evidence of mild neurological abnormalities in muscle tone that could suggest minor neurological dysfunctions (MND), such as “neurological soft signs” (NSS; Shaffer et al., 1985; Hadders-Algra et al., 2009) or neuromotor disorders with mild cerebral palsy (CP). CP could be a continuum of DCD according to Pearsall-Jones et al. (2010). Thus, the term “neurological soft sign” as applied to minor neurological dysfunction is usually intended to reflect a typically non-normal performance, without evidence of focal neurological involvement (Hadders-Algra et al., 2009), on various psychomotor or somatosensory tasks, such as dysdiadochokinesia, synkinesia, tactile localisation deficits, motor speed, mild dysfunction in muscle tone regulation, and asymmetric reflexes (Shaffer et al., 1985). Neurological soft signs normally decline with the child's age thus evidencing the progressive maturity of the nervous system. However, NSSs and MND are not clear-cut in the literature and often used similarly, and the different studies do not use the same sensori-motor milestones. Although, we can consider that MND (mainly covered by basic tests of sensory function, including NSS, fine and gross motor control, postural control, dyskinesia, associated movements, and neuromotor signs of mild spasticity) evidence impairment of the motor pathways with asymmetric reflexes or phasic stretch reflex (PSR; see Amiel-Tison et al., 1996) never investigated in DCD studies.

The few studies that identified CP with risk factors for DCD without known neurological involvement suggest that CP is strongly related to preterm birth or perinatal risk factors (Foulder-Hughes and Cooke, 2003; Hadders-Algra et al., 2009; Lingam et al., 2009; Pearsall-Jones et al., 2010).

However, the neuro-anatomical origins of DCD in children born full-term are probably different and not clearly understood (see Ahonen et al., 2004; Zwiker et al., 2009; Vaivre-Douret et al., 2011a; Vaivre-Douret, 2014), and cerebral involvement in DCD children with MND is not systematically explored.

### Aims of the current study

The purposes of the present study aims was to explore multivariate associations between DCD subtypes and several neuropsychomotor, psychological, and neuropsychological features, in order to:

1. refine the discriminant features of DCD subtyping markers in the MX group, and
2. highlight specific co-morbidities, such as MND with NSS or mild distal spasticity, almost never investigated in DCD children, in the absence of other features of known permanent or transient neurological disorders (Lyytinen and Ahonen, 1988; Vaivre-Douret et al., 2011b).
3. understand the nature and etiology of the various types of pathophysiology in DCD.

Data from a standardized physical developmental instrument (NP-MOT), comprising (qualitative and quantitative) validated rating scales, and age-specific developmental examinations are analyzed.

## Methods

### Participants

A sample of children was selected from those consecutively referred to the out-patient consultation of the Department of Paediatrics, Cochin Port-Royal Hospital, and the department of Child Psychiatry, Necker Hospital, Paris, France, on the basis of DSM-IV criteria, which are similar to DSM-5. Clinical history and the Geuze questionnaire (2005) were used for criteria A and B in accordance with the European Academy for Childhood Disability recommendations (Blank et al., 2012). The Institutional Review Board of Paris Descartes University ethics committee approved our study to collect data from participant assessments (IRB: 20134900001072), undertaken in accordance with the Declaration of Helsinki. Participants provided written informed consent before the start of the study, signed by a parent or legal representative before each child was enrolled into our study. Inclusion criteria were strict. Children had not been assessed previously and were not taking medication or having occupational /physical therapy. Children with attention deficit hyperactivity disorder, sensory deficit, psychiatric, and general medical abnormalities or traumatic brain injury were not included, nor was any child born premature (<37 weeks).

### Procedure

Data from children aged between 6 and 13 years were eligible for inclusion in this study. Children recruited were assigned to the two DCD subtypes validated in a previous study (Vaivre-Douret et al., 2011a; Lalanne et al., 2012), that is to say to a visual spatial and constructional (VSC) subgroup or to a Mixed subgroup (MX). The ideomotor subgroup was excluded from this study because cases were rare and because the group was already well identified in the study by Vaivre-Douret et al. (2011a).

All children completed a standard measure of intelligence, the Wechsher Intelligence Scale for children according to the age (WPPSI-R or WPPSI-III, WISC-III or WISC-IV). Verbal (VIQ), Performance (PIQ), and Total IQ (TIQ) scores were expressed as standardized scores (mean 100, *SD* = 15).

Data about pregnancy and delivery, age of early motor acquisitions (sitting alone, walking), any difficulties with constructional manipulatory play, such as puzzles and Lego blocks following a model, school performances in basic skills, as attested by tests, (spelling/reading, arithmetic, and writing) were also collected.

All children were assessed with standardized tools described in a previous study (Vaivre-Douret et al., 2011a; Robert et al., 2014), they performed neuropsychomotor physical tasks in the NP-MOT battery (Vaivre-Douret, 2006) with assessment of MND exploring NSS, and a neuropsychological evaluation of all brain functions.

The age-standardized child assessment using the French NP-MOT test battery (Vaivre-Douret, 2006) is applicable to children as young as 4 years. It has been found to have adequate test-retest reliability and internal consistency. Correlation coefficients of the NP-MOT with the BOTMP (Bruininks, 1978) range from 0.72 to 0.84, for motor coordination and balance.

The NP-MOT battery enables physical assessment of passive/active muscular tone of limbs and axial tone, highlighting NSS denoting the existence of MND, such as limb pyramidal dysfunction, completed by the assessment of basic motor function, control and regulation in gross motor tasks, gait, balance, coordination, manual dexterity, praxis, gnosopraxis (non-meaningful hand and finger imitation of gestures), digital perception, laterality, bodily spatial integration, rhythmic, and auditory attention tasks (see details of components in Table 1). The exploration of MND was similar for some components and tasks to that using the Touwen Infant Neurological Examination (Touwen, 1979) but more similar in scoring to the Quick Neurological Screening Test (QNST of Muttey et al., 1978) or the Physical and Neurological Examination Soft Sign Scale (PANESS, (Denckla, 1985). The developmental NP-MOT assessment is standardized with each subtest and milestone scored from qualitative and quantitative viewpoints, with each score converted to a standard deviation vs. mean, based on normative data for age and applicable to children as young as 4–8 years and 6 months old. There is a saturation of the scores from 8 years, allowing the use of the NP-MOT for older children or adults.

**Table 1:** **Standardized developmental assessment battery of neuro-psychomotor functions in children**.

**PART 1 OF NP-MOT BATTERY**				
**Neuromuscular tone examination**	**Gross motor**	**Laterality**	**Manual praxis**	**Digital perception (gnosis)**
**Passive muscular tone**: Amplitude (degrees) and resistance R/L scores (Hypotonic angle/hypertonic) • Dangling of wrist • Dangling of foot • Extensibility of shoulder • Extensibility of wrist • Extensibility of popliteal angles • Extensibility of adductor angles • Extensibility of heel-ear angle • Extensibility of trunk (extension/flexion) • Extensibility of foot dorsi-flexion angle and rapid/slow stretch reflex (looking for abnormality: phasic/tonic stretching) • Passive mobilization of members (relaxation) • Knee jerk reflex (R/L) **Active muscular tone**/dysdiadochokinesis and synkinetic movements score (co-movements and mirror movements/axial, ipsilateral, other side): • Rapid pronation and supination of the hand (R/L) • Repeated opening and closing of both hands • Repeated opening and closing the mouth **Sitting tone** • Sit on the floor: pushes right left, backwards/forwards **Standing tone** • Feet together: 3 light pushes on the thorax Observation of the tibiallis anterior muscle contraction	**Dynamic balance** Timing/quality's score of upper and lower limbs • Spontaneous walk • Walk on a line heel-toe and backwards (≥6 steps) • Walking on tiptoes (≥6 steps) • Walking on heels (≥6 steps) • Jump from a podium feet together • Score of coordination between limbs and for postural control **Static balance** ≥ 10 s Timing/quality's score of limbs • Feet together • On one foot • On tiptoes	**Spontaneous gestual laterality of upper limbs (R/L)** • With arms extended on either side, cross one arm over the other • With clenched fists and elbows bent, place one fist over the other • Arms outstretched forwards with hands open, place one hand over the other • Pointing the index finger of each hand, with elbows bent, place one index finger over the other **Usual laterality of upper and lower limbs/preference** Hand (R/L, R = L) • Putting a string through a flower on a card to form the stem • With a (fake) box of matches, strike a match • Rub out a cross in the middle of a page Foot (R/L, R = L) • Kick a ball (four trials) • Homogeneity of usual dominant laterality between upper/lower limb: yes/no **Director eye dominant** (R/L, R = L) • Test for dominant eye: using 2 hands, place the large part of a cone over the two eyes (only the dominant eye is visible for the investigator at the small part of the cone) **Psychosocial laterality** (R/L) score of hand preference 4/6 milestones Representations of transitive/intransitive gestures on verbal command Observation of the dominant hand and the quality (primitive/ symbolic) of the gesture for the following: • Throwing a ball with one hand • Hammering a nail • Opening a door with a key • Brushing one's teeth with a toothbrush • Brushing one's hair • Eating soup with a soup spoon	**Bimanual coordination: 10 pronation-supination movements** (Timing and quality scores) • Symmetrical movements (synchronization) • Asymmetrical movements (synchronization) **Digital praxis** (Timing and quality scores) • Index finger-thumb (R/L timing on 20 movements) • Successive touching thumb to fingertips (R/L timing on 20 movements) **Gnosopraxis imitation of gestures** (Vaivre-Douret, 2002) Score of gesture quality with mirror movement: immediately/imitation step by step • 10 Hand gestures • 16 Finger gestures **Representational gestures**: transitive/intransitive gestures on verbal command (see psychosocial laterality with the score of the quality of gestures) • Note each milestone (6), excecuted with primitive/Symbolic gestures **Oro-facial praxis**: Facial, tongue, and mouth praxis • Positioning mouth to whistle or blow • Puffing out the cheeks • Pulling out the tongue toward the chin • Pulling out the tongue to the right • Pulling out the tongue to the left • Pulling out the tongue toward the nose • Making a galloping noise (clicking the tongue) **Dressing skills**: (Information asked from the parents) • Puts his/her clothes on correctly • Confuses body segments • Puts clothes on inside out • Confuses left and right, or back and front • Difficulties in fine motor skill (buttoning-up)	**Localization of digital tactile stimuli** • Right hand (10 fingers stimuli) • Left hand (10 fingers stimuli)
		**Tonic laterality** (see tone examination/resistance corresponding to dominance side) Upper limbs ((homogeneous dominance on 3 milestones = afirmed, 2 = not affirmed/ indeterminate • Dangling of wrist • Extensibility of wrist • Extensibility of shoulder Lower limbs (homogeneous dominance on 3 milestones = afirmed/ 2 = not affirmed/ indeterminate • Dangling of foot • Extensibility of foot • Standing tone Homogeneity of tonic dominant laterality between upper/lower limbs (normaly crossed) **Dominance of functional laterality** (Spontaneous gestual + Usual + Psychosocial) 3 milestones R or L = affirmed laterality, 2 = not affirmed, indeterminate if at least 2 tasks R = L		
**PART 2 OF NP-MOT BATTERY**				
**Manual dexterity**	**Bodily spatial integration (R/L)**		**Rhythmic tasks**	**Auditory-attentional task**
**Putting the row of twelve counters one by one into a box** (12 rows) • Right hand score of timing and quality • Left hand score of timing and quality • Dominant score (timing L - timing R divised by the fastest hand/R/L	**Score in relation to self** (hesitation > 3 s/immediately) • Pointing (four items) • Verbal command with axial crossing gesture (four items) **Score knowing left from right** (hesitation > 3 s/immediately) • Pointing on the examiner (two items) • Pointing on a doll (two items) • Imitation of the examiner with axial crossing gestures (eight items; used from 5,9 years old) **Score with regard to the objects** **(R/L)** (hesitation > 3 s/immediately) • Two objects • Three objects **Map direction (R/L)** used from 5,9 years old **Global score of bodily spatial integration**		**Spontaneous rate of regular hand taps** • On 21 taps /timing and gestual regularity **Auditory-visual-kinesthetic adaptation task via imitation of examiner tapping patterns** • Imitation of hands (two items) • Imitation of feet (two items) • Imitation of hands and feet (two items) **Auditory-perceptual-motor rhythmic adaptation with metronome speed at 90, 60, and 120** • Claps (synchronization ≥ 6 s or ≥ 15 s) • Walk (synchronization ≥ 6 s or ≥ 15 s)	**Series of 16 taping with a chopsticks** (timing and quality scores) • Taps in a go/no-go (if 1 tap/2 taps and inversely 2 taps/1 tap)

*(NP-MOT, Vaivre-Douret, 2006): components of the examination in bold, based on normative data for age (qualitative and quantitative measures)*.

Special attention was paid to the presence of MND in the NP-MOT battery, along with NSS and neuromotor signs such as the presence of lower limb pyramidal tract dysfunction like phasic stretch reflex (PSR) in one or both gastrocnemius muscles, but normal Babinsky's reflex. Indeed, PSR is systematically assessed in distal muscle tone examinations of the lower limbs as a sign of mild spasticity evidencing impairment of the motor pathways (Amiel-Tison et al., 1996): fast dorsiflexion of the foot with the lower limb extended from the knee is arrested by resistance to the passive movement, but the movement can be completed. This response is known as “phasic stretch reflex” (Amiel-Tison et al., 1996). It is known that PSR appears at 6–18 months, uni- or bi-laterally, and, when present, persists throughout life (Amiel-Tison et al., 1996).

Data from neuropsychological standardized assessments (See Table 2) were collected as previously described in other studies (Vaivre-Douret et al., 2011a; Robert et al., 2014). They concerned visual-motor integration, visual-perceptivo motricity (constructional and visuo-spatial structuring), visual perception, visuo-spatial attention, executive functions, language, visuo-perceptive functions, neurovisual examination with smooth visual pursuits and results of brain MRI.

**Table 2:** **Others clinical investigations**.

**AMNESIA**
- Few questions to parents about age at the time of the first motor acquisitions (i.e., sitting alone, crawling, walking alone, first sentences), medical history, visual refraction disorder, difficulties with constructional manipulatory play, such as puzzles and Lego blocks following a model, and academic performances (arithmetic, reading) noted in the school reports.
- Geuze's questionnaire (2005)/for criteria A and B in DSM.
**NEUROPSYCHOLOGICAL EVALUATIONS**
- A standard Wechsler measure of intelligence (WPPSI-R or WPPSI-III, WISC-III or WISC-IV).
- Visual constructional skills (Khos block design).
- Visual-spatial structuring (copying Rey's complex geometric figure).
- Beery's Visual-Motor Integration test with copying of 2D geometric graphic representations.
- A handwriting scale was also used to detect dysgraphia (de Ajuriaguerra) similar to BHK.
- Visual-spatial attention (bell-crossing test Odédys).
- Visual perception using form recognition tasks (Frostig), tangled lines and visual gnosia with outlines of animals, outlines of muddled fruits.
- Kinaesthetic perception (with status memory test assessed by positioning the child's arm and finger and asking him with eyes closed to remember and repeat.
- Auditory memory and working memory tasks (digit span).
- Visuo-spatial memory (Rey's complex geometric figure).
- Mental planning executive functions (Porteus Labyrinth and Tower of London test).
- Language screening battery (Odédys, Neel) included tasks of reading, repetition of words, and logatoms, picture-naming speed, meta-phonological tests.
**NEUROVISUAL EXAMINATION**
- Electro-retinogram (ERG).
- Visually evoked potentials (VEP).
- Motor electro-oculogram (vertical and horizontal pursuits).
**MAGNETIC RESONANCE IMAGING**
- Anatomical MRI was performed with a 1.5 Tesla (Signa General Electric).

*All test scores were standardized following authors' scoring guidelines and developmental norms*.

### Statistical analysis

Tree-based bagged classifiers were used in the current study. These statistical multivariate models belong to the family of “ensemble methods” which combine estimates gathered from various models, by averaging a collection of decisions from weak classifiers. Unlike the classic Random ForestTM algorithm (Breiman, 2001), multivariate classifiers like “logic forest” were developed to explore interactions of various orders between binary markers. They can also be used to perform so-called feature selection and to identify explanatory variables predictive of an observed clinical diagnosis. These classifiers may or may not include an extra bagging step (Schwender and Ickstadt, 2007; Wolf et al., 2010). This makes these algorithms more likely to uncover meaningful associations between clinical records in relation to discrete diagnostic classes when their number is too large to allow for classic logistic regression.

Only VSC and MX children were considered in the classification procedure. A total of 44 binary variables or tasks were considered. Tasks were scored 0 (success) and 1 (failure) based on percentile or standard deviation (below 1 *SD* or 10th percentile, depending on the test) in accordance with standardized instructions and developmental norms. Analysis of continuous outcomes according to one or more classification factors was performed using parametric ANOVA, and Pearson's chi-square tests were used to analyse two-way cross-classification between qualitative variables. Two-group comparisons of scale scores were performed with *t*-tests. A fixed Type I error rate of 5% was retained for all statistical tests.

The identification of relevant interactions relied on a logic regression model (Schwender and Ickstadt, 2007) which is applied iteratively on bootstrap samples of the original data set, considering all logical combinations of markers, which then enables a measure of the relative importance of variables or combinations thereof to be obtained, based on the out-of-bag observations. The bagged logistic regression classifier (Schwender and Ickstadt, 2007; Wolf et al., 2010) was calibrated on a training sample (*N* = 42, 72%), stratified for clinical diagnosis, and its classification accuracy was assessed on an independent validation sample (*N* = 16, 28%, including 9 VSC and 7 MX). The tuning of the hyperparameters of the model (number of leaves—2 or 3, and number of trees—100, 300, or 500) was performed using bootstrap resampling (25 runs), and the hyperparameters that optimized classification accuracy on the training sample. In addition to performing univariate and multivariate feature selection, the Logic Forest classifier also ranks the variables in relation to their interactions. In this case, the following notation was used: & denotes logical intersection (“and”), and ¬ denotes logical negation (“not”). All measures of “importance of variables” (defined as the number of out-of-bag cases correctly classified) (Ruczinski et al., 2003) for univariate and interacting features were standardized on a 0 to 1 scale by rescaling individual measures on the basis of the highest-ranking variables.

The reference category used was VSC so that this classifier selects the best features and feature interactions to predict the MX class.

R statistical software was used for all analyses.

## Results

A total of 58 children (mean age 8.8 years, *SD* = 2.5) met all inclusion criteria, among whom 33 were classified VSC (57%) vs. 25 MX DCD (43%). Demographic information for all study participants is provided in Table 3. The sample was mainly composed of boys (83%) average age 9 (*SD* = 2.5), with full IQ in the expected range. The following instruments were used for IQ assessment: WISC-III (*n* = 38, 61%), WISC-IV (*n* = 10, 16%), WPPSI-R (*n* = 11, 18%), and WPPSI-III (*n* = 3, 5%). Verbal and performance IQ scores were significantly higher (respectively, *p* = 0.001 and *p* = 0.002) in the VSC group (VIQ = 116; *SD* = 19.6, PIQ = 95.5; *SD* = 19.8) than in the MX group (VIQ = 96; *SD* = 25.1, PIQ = 80; *SD* = 22.1).

**Table 3:** **Children's characteristics**.

**Variable**	**Training**	**Validation**	**All children**	
**DEMOGRAPHIC INFORMATION**				
Gender (Male)	78% (36)	94% (16)	83% (52)	
Age (years)	8.5 (2.1), 6.8–9.7	9.5 (3.2), 6.6–12.3	8.8 (2.5), 6.810.4	
Full IQ	98 (22), 85–114	106 (24), 91–121	100 (23), 86–115	
Performance IQ	89 (21), 73–102	93 (22), 75–107	90 (21), 74–105	
Verbal IQ	106 (21), 92–122	114 (26), 100130	108 (23), 92–124	
**LEARNING DISORDERS AND CEREBRAL ABNORMALITIES**				
**Reading/Spelling**				
MX	44% (8)	29% (2)	40% (10)	*P* = 0.206
VSC	17% (4)	33% (3)	21% (7)	
**Arithmetic**				
MX	100% (18)	100% (7)	100% (25)	*P* = 0.069
VSC	79% (19)	89% (8)	82% (27)	
**MRI abnormalities**				
MX	39% (7)	57% (4)	44% (11)	*P* = 0.751
VSC	42% (10)	22% (2)	36% (12)	
**Phasic stretch reflex (PSR)**				
MX	22% (4)	71% (5)	36% (9)	*P* = 0.861
VSC	25% (6)	44% (4)	30% (10)	

*Categorical variables are summarized with proportions (counts), and means (SD) for numerical variables and interquartile-ranges are provided*.

*VSC, Visuo-spatial/visuo-constructional DCD subtype; MX, Mixed DCD subtype; MRI, magnetic resonance imaging*.

Regarding learning disabilities, all MX children were impaired in arithmetic. None of the children had significant psychiatric or medical history.

We detected similar proportions of phasic stretch reflex (PSR), never previously highlighted during child development, with 30% in the VSC group vs. 36% in the MX group.

There were 44% abnormal MRI scans among the MX subtype children (vs. 36% abnormal MRIs among VSC children). The MRI scans were heterogeneous and non-specific to subtype, e.g., multiple punctate white matter hyperintensities and dilated Virshow-Robin spaces, ventricular dilatation, small hippocampus, non-specific cysts, periventricular white matter abnormalities, dysmorphism of the corpus callosum.

### Feature selection for DCD subtype

**Figure 1:**
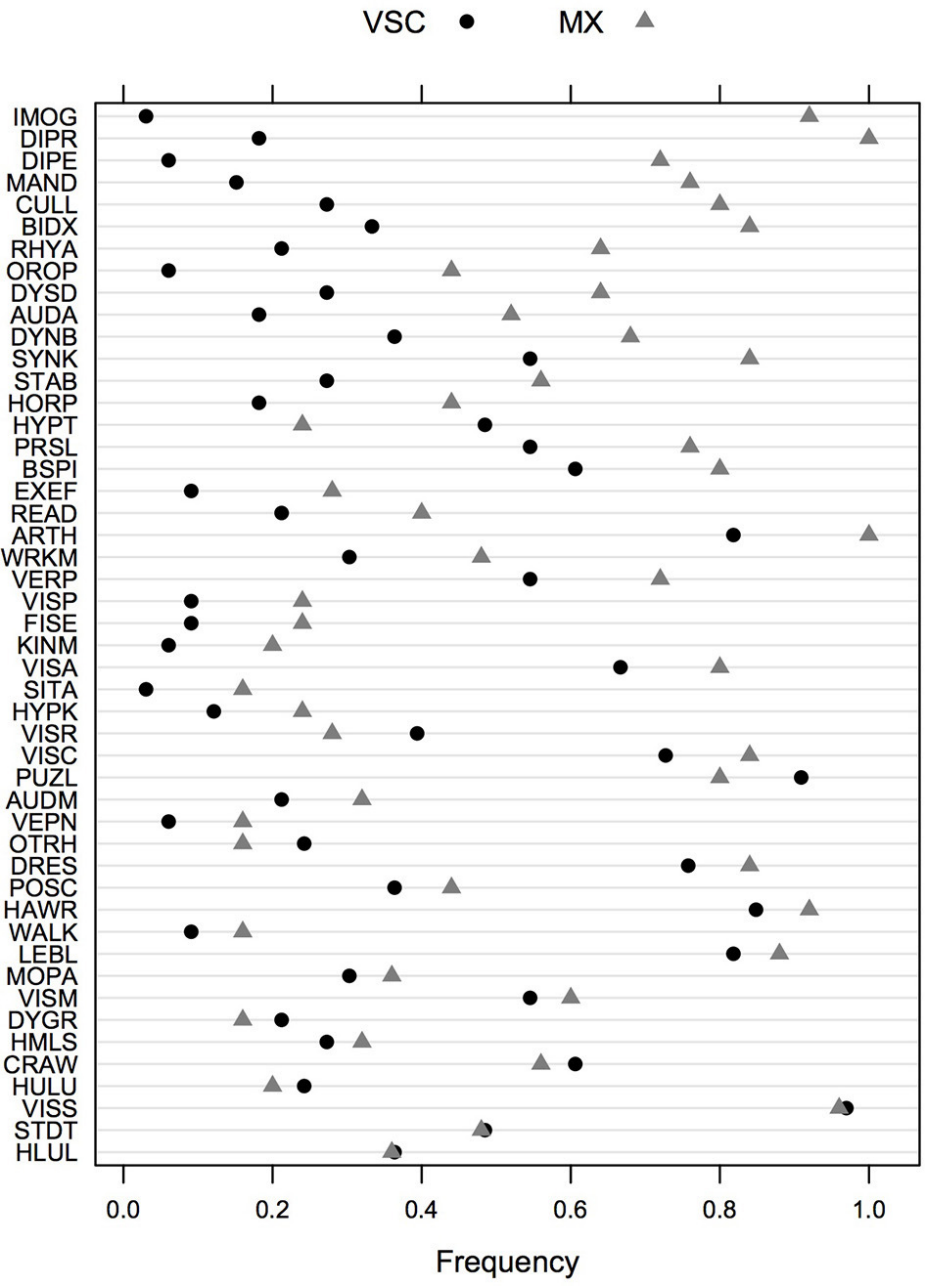
**Frequency of impairments in VSC and MX patients for the whole test battery**. Items are ordered in decreasing order of absolute difference between the two groups. VSC, Visuo-spatial/visuo-constructional subtype DCD; MX, Mixed subtype DCD.

The frequency of impairment for the two DCD groups of children (MX and VSC) is shown in Figure 1, sorted by decreasing (absolute) value of the difference between the two groups. Univariate screening for predictors of interest suggests that imitation of gestures (92% in MX children vs. 3% in VSC children), digital praxis (100% vs. 18%), digital perception (72% vs. 6%), manual dexterity (76% vs. 15%), and coordination between upper and lower limbs (80% vs. 27%) are among the most discriminant features in DCD for MX subtype.

Regarding model performance, classification accuracy was estimated at 88% on the training sample. Predictive performance was perfect on the validation sample, where VSC had an observed prevalence of 0.56.

**Figure 2:**
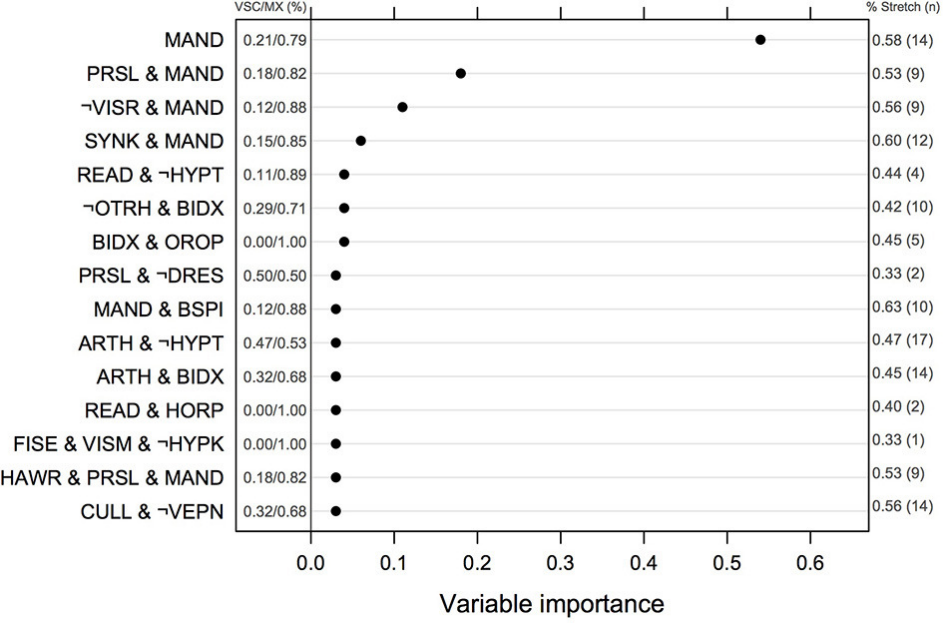
**Normalized measures of variable of importance from the Logic Forest classifier on the full sample (*N* = 58)**. The frequency of impairment for MX and VSC children is shown on the left part of the figure. The symbol ¬ indicates logical negation, which means no failure in this case. VSC, Visuo-spatial/visuo-constructional subtype DCD; MX, Mixed subtype DCD.

The ranking of univariate and interacting features for the Logic Forest classifier without bagging is shown in Figure 2. The number of MX and VSC cases fulfilling each criterion or combination thereof is reported to the left of the vertical line centered on 0. Manual dexterity appeared to be a relevant predictor, either alone (out of the 24 children failing this task, 19 were MX subtype) or in combination with other variables (e.g., slowness of praxia, synkinesis, bodily spatial integration, and handwriting). Other important learning disorders included reading and arithmetic. It is worth noting that the first five combinations of variables were found on several independent runs of the same classifier, suggesting they are quite reliable indicators of clinical typology.

Results from logistic regression with bagging indicated that the most important variables or combinations of variables were (in decreasing order of importance): imitation of gestures, arithmetic and imitation of gestures, digital praxia, imitation of gestures and digital praxia, and digital praxia and digital perception. Univariate features that were identified by this classifier are summarized in Table 4.

**Table 4:** **Frequency of impairment for the 10 most important predictors selected by the Logic Forest classifier, according to phasic stretch reflex status, and subtype of dyspraxia**.

	**All**		**VSC**		***p-value***	**MX**		***p-value***
	**No stretch**	**Stretch**	**No stretch** **(*N* = 23)**	**Stretch** **(*N* = 10)**		**No stretch** **(*N* = 16)**	**Stretch** **(*N* = 9)**	
Imitation of gestures	0.385	0.474	0.043	0.000	1.000	0.875	1.000	0.518
Digital praxis	0.462	0.684	0.087	0.400	0.060	1.000	1.000	0.219
Digital perception	0.282	0.474	0.043	0.100	1.000	0.625	0.889	0.210
Manual dexterity	0.256	0.737	0.000	0.500	**0.001**	0.625	1.000	0.065
Arithmetic	0.846	1.000	0.739	1.000	0.128	1.000	1.000	0.233
Coordination upper/lower limbs	0.333	0.842	0.087	0.700	**0.002**	0.688	1.000	0.133
Reading/spelling	0.308	0.263	0.261	0.100	0.398	0.375	0.444	1.000
Visual spatial attention	0.667	0.842	0.652	0.700	1.000	0.688	1.000	0.127
Orofacial praxia	0.205	0.263	0.087	0.000	0.577	0.375	0.556	0.437
Vertical pursuit	0.564	0.737	0.435	0.800	0.079	0.750	0.667	1.000

*VSC, Visuo-spatial/visuo-constructional DCD subtype; MX, Mixed DCD subtype. p-values in bold are significant p-values*.

### Relationship between motor pathway disorder (PSR) and DCD subtype

The phasic stretch reflex (PSR) was observed in one case out of three (19/58, 33%) and it was not specific to the DCD subtype [X^2^(1) = 0.03, *p* = 0.861]. PSR was mainly lateralized on the left (53%, *n* = 10), or on left and right (47%, *n* = 9 including 4 MX and 5 VSC).

Imbalance in passive axial tone in favor of extension (17/58, 29.3%), correlated to PSR in 17/19 cases [89.4%, X^2^(1) = 49.36, *p* < 0.001], making PSR a marker for motor pathway disorder. VSC children exhibiting PSR disorder were significantly more likely to be impaired in tasks involving coordination between upper and lower limbs (70%, *n* = 7) in comparison to those free from any such disorder (9%, *n* = 2) [X^2^(1) = 10.3, *p* = 0.001]. In MX children, this effect was less pronounced (100 vs. 69%).

The associations between phasic stretch reflex (PSR) and the top 10 univariate features identified by the logistic classifier are given in Table 4, for all children, for VSC and MX children separately. Subgroup comparisons using chi-square tests with *p*-values computed by Monte Carlo simulation suggest that none of the variables showed a significant association with PSR in MX children, whereas impairments in manual dexterity (*p* = 0.001, not corrected for multiple testing) and in coordination between upper and lower limbs (*p* = 0.002) were more likely to be found in VSC children presenting a motor pathway disorder. Apart from variables selected by this classifier, PSR in MX children was mainly associated with MND, namely sitting tone (89% impairment, *p* = 0.004) and dysdiadochokinesia (100% impairment, *p* = 0.011). For these variables, VSC children with PSR were impaired in 50 to 70% of cases. Finally, counts and frequencies for children with PSR are shown in Figure 2 for the top-ranking discriminant combinations of variables.

### Relationship between phasic stretch reflex (PSR) and other variables

For the 38% with medical complications at birth, there was no association with PSR [X^2^(1) = 1.75, *p* = 0.186; 4/9 MX and 6/13 VSC]. Children with PSR were more likely to have been able to sit alone at the expected age plus or minus one SD (*n* = 12, 63%) compared to the other children [X^2^(1) = 6.13, *p* = 0.013], while there was no significant difference related to presence of PSR for walking [X^2^(1) = 2.25, *p* = 0.133]. More children with PSR were late walkers (69 vs. 50% without PSR).

No significant associations were found between PSR and dysgraphy [X^2^(1) = 2.93, *p* = 0.087], hand-writing [X^2^(1) = 2.37, *p* = 0.124; 37% with PSR], arithmetic [X^2^(1) = 3.26, *p* = 0.071], language [X^2^(1) = 0.12, *p* = 0.727], vertical pursuit [X^2^(1) = 1.86, *p* = 0.394], horizontal pursuit [X^2^(1) = 3.20, *p* = 0.202], auditory (*p* = 1) or visual spatial attention [X^2^(1) = 1.19, *p* = 0.278], or working memory [X^2^(1) = 0.21, *p* = 0.648]. However, there was a significant association between PSR and dysdiadochokinesia [X^2^(1) = 17.06, *p* < 0.001], and executive functions [X^2^(1) = 5.70, *p* = 0.017; 70% with PSR, 5/7 MX, and 2/3 VSC], but not synkinesia [X^2^(1) = 1.06, *p* = 0.304]. Regarding IQ levels, children presenting PSR had lower average full IQ (91.9, *SD* = 23.1) compared to children without PSR (103.4, *SD* = 23.0, Welch *t*-test, *p* = 0.084). They had lower verbal (98.9 vs. 112.3, *p* = 0.047) and performance IQ scores (81.5 vs. 92.7, *p* = 0.055).

Usual laterality was poorly established in 12% of cases (*n* = 7, 4 MX, and 3 VSC) for the lower limbs, and in 22% of cases (*n* = 13, 4 MX, and 9 VSC) for the upper limbs. However, these frequencies increased for children with phasic stretch reflex (6/7 and 6/13, respectively, with three children exhibiting lateralization problems for both upper and lower limbs).

Evaluation of tonic laterality of the upper limbs showed a tonic dominant limb on one side that was not correlated with the side of usual laterality as would be normal, and also no tonic difference between the two upper limbs when PSR was present [X^2^(2) = 6.55, *p* = 0.038].

There were 24% left-handed children (*n* = 14), without increase in dysgraphia disorder (2/14) compared to right-handed children (8/43).

No association between abnormal MRI and phasic stretch reflex [X^2^(1) = 0.30, *p* = 0.581], or dysdiadochokinesis and synkinesis (*p* = 1), or complications at birth (*p* = 1) was found.

## Discussion

In this study, results for a sample of children born full-term, affected by DCD and submitted to a complete battery of neuropsychological, neurodevelopmental psychomotor function standardized assessments, including MND, with developmental normative data for age (Vaivre-Douret, 2006), made it possible to identify salient DCD markers in a global group of DCD (MX group) and to highlight specific neurodevelopmental co-morbidities.

The MX group has long been shown to define a clear-cut category in previous studies, with high levels of motor impairment in fine and global motricity (Lyytinen and Ahonen, 1988; Lundy-Ekman et al., 1991; Dewey and Kaplan, 1994; Hoare, 1994; Miyahara, 1994; Wright and Sugden, 1996; Macnab et al., 2001; Green et al., 2008; Vaivre-Douret et al., 2011a; Wilson et al., 2013), although authors do not agree on a common etiology.

It is important to note, however, that few researchers used both motor and perceptivo-motor measures (Lyytinen and Ahonen, 1988; Lundy-Ekman et al., 1991; Dewey and Kaplan, 1994; Hoare, 1994; Macnab et al., 2001; Green et al., 2008; Vaivre-Douret et al., 2011a), and only some studies implemented non-meaningful hand and finger positions in gestural imitation assessment (Dewey and Kaplan, 1994; Green et al., 2008; Vaivre-Douret et al., 2011a). In addition, the cluster analyses did not include neurological developmental dysfunction (i.e., NSS or MND), which might inform on the nature of the developmental motor disorders.

Our findings show that MX children were significantly impaired when asked to imitate non-meaningful gestures, in digital praxis and digital perception (specific impairments of Ideomotor DCD, see Lalanne et al., 2012) and in tasks specifically involving manual dexterity and coordination between upper and lower limbs.

The present study is, to our knowledge, the first report a complete investigation of clinical developmental parameters on a DCD sample. Indeed, there are above all studies on subtypes of DCD looking for differences among DCD children in their performances in the predictive control of action and for sensory-perceptual dysfunction, using chronometric and neuropsychological measures (see, Wilson et al., 2013). However, these studies do not take co-morbidities into account and qualitative and quantitative measures of the performance in the result of the motor performance score contrary to the NP-MOT battery (Vaivre-Douret, 2006). Other research looks at relationships between learning disabilities and MND entailing neuromotor abnormality (such as CP) and DCD, but does not distinguish the subtypes of DCD (Lyytinen and Ahonen, 1988; Hadders-Algra et al., 2009; Pearsall-Jones et al., 2010). In addition, MND are often confused with neurodevelopmental sensory-motor functions. In fact, the term MNS, as it relates to neurological dysfunction, is usually defined as minor abnormalities in the standard neurological examination (tone, reflexes…), in the absence of focal or transient neurological disorder. MND is used to refer to atypical performances on various somatosensory tasks, with heterogeneous assessments implemented between studies, such as the PANESS examination (Denckla, 1985) covering gait, stance, laterality, quality of rapid movements, impersistence, involuntary movement, repetitive speed of movement, and sequenced speed of movement, asymmetrical movement), or the protocol devised by Shaffer et al. (1985) including stereognosis, graphaesthesia, dysdiadochokinesic mirror movements, motor speed, and involuntary movements.

The present findings are unexpected in that they evidence a high incidence of a motor pathway dysfunction (evidenced by mild spasticity of gastrocnemius muscles in the lower limbs) in 33% of the children. This frequent abnormality in the VSC and MX groups, on the left side (53%) or bilateral (47%), suggests that the involvement of the right cortex could undepin visuo-spatial motor problems. In our sample, phasic stretch reflex (PSR) is correlated with MND, such as hyper-extensibility of axial tone, dysdiadochokinesia or disturbed sitting tone, highlighting involvements in the motor area of the cerebral cortex.

The results show that PSR is significantly associated in either subtype (VSC or MX) with marked impairment of upper and lower limb coordination, and with manual dexterity tasks. There is a risk of concluding to an increase in these impairments (in terms of frequency), especially in the VSC subtype, because usually this subtype is more specific to visual-spatial motricity and visual motor integration (Lalanne et al., 2012). When these impairments are associated in VSC subgroup it is mainly because PSR generates hemiparesia affecting the left side of the body, suggesting right hemispherical disturbance.

These two markers of gross motor and fine dexterity are often described in DCD cluster studies as a subgroup with deficit in all the motor skills. But it is never observed that it could originate from a specific impairment of motor execution, probably because examination of muscular tone and neuromotor examination are rarely performed in such studies. Thus, the fact that this is more marked in VSC children is unexpected according to the diagnostic criteria for VSC subtype of DCD described in previous studies (Vaivre-Douret et al., 2011a; Lalanne et al., 2012). Thus, the presence of phasic stretch reflex (PSR) appears as a co-morbid impairment of motor execution, increasing impairment of gross and fine motricity, and this could explain why DCD appears as a collection of motor disorders in a heterogeneous group in numerous studies on DCD (Hadders-Algra et al., 2009). PSR is a consequence of a developmental motor dysfunction of the pyramidal tract. It indicates an impairment of voluntary movement from the premotor cortex. The pathophysiological interpretation could be a mild form of cerebral palsy on the corticospinal tract, which means a disturbance in the motor pathways and hence impaired control of the motor neurones. This could be found in etiological contexts other than DCD. The fact that PSR was found to correlate with imbalance of passive axial tone with excessive dorsal extension of the trunk and excessive osteotendinous reflexes suggests that it is accounted for by the same mechanism of higher control that is impaired in mild lesions of the cerebral hemispheres resulting in the de-inhibition of lower structures (Amiel-Tison et al., 1996). This neuromotor abnormality should be detected during clinical pediatric examinations before 24 months of life (Amiel-Tison et al., 1996) but this minor neurological dysfunction has never been systematically investigated because it is a discreet clinical distal abnormality.

Furthermore, in MX children, phasic stretch reflex can be significantly associated with features of orofacial praxia, involving impairment of orofacial motricity from the somatotopy of the cortical area in the motor homonculus representing the face and the mouth.

Although the features highlighted by the multivariate classifier are comparable to those discussed in Lalanne et al. (2012) but it should be noted that PSR was not included in the set of contributing features in this study. This means that the highest-ranking variables (imitation of gestures, digital praxis, etc.) remain important predictors of the Mixed DCD subtype, even if PSR is not included in the classification.

However, while we found learning disabilities, often described (Visser, 2003; Vaivre-Douret et al., 2011a; Wilson et al., 2013; Vaivre-Douret, 2014), in reading or in mathematics, these features, others concerning handwriting or dysgraphia, language and smooth pursuit (Robert et al., 2014), and visual and auditory attention or memory problems, are not associated with PSR.

As PSR is a motor pathway dysfunction arising from the motor cortex (Prefrontal), it is not surprising that it is significantly associated with impairment of executive functions in the frontal control area. Thus, the etiology of DCD is often compared to adult apraxia resulting from brain damage in the left parietal lobe and in the premotor frontal cortex.

The etiology of DCD appears confused on account of the umbrella term of motor dysfunction. However, some studies (Lundy-Ekman et al., 1991; Visser, 2003; Vaivre-Douret et al., 2011a,b, 2015) have pointed to the implication of the subcortical network of the brain. Indeed, incorrect information is sent to the cortex (prefrontal, parietal, temporo-occipital) resulting in disturbances in motor planning and programming of movement that cannot be automatically corrected because of a dysfunction of the cerebellum-thalamus-basal ganglia circuit. Using functional magnetic resonance imaging in a recent study of Zwicker et al. (2011), it has been demonstrated that there is under-activation in the cerebellar–parietal and cerebellar–prefrontal networks and in brain regions associated with visual-spatial learning. Our findings suggest a dissociation of the causal origins of motor disorders between brain impairments from the cortex (i.e., pyramidal tract lateral cortico-spinal affecting the motor command of distal motricity) vs. those in the subcortical basal ganglia region (i.e., dysdiadochokinesia, regulation and control of movement…) and in thalamus (i.e., bodily integration with imitation of finger gestures) and the cerebellum (timing of movement, rhythmic adaptation, …). However, these disorders may be associated, possibly explaining more marked co-morbidity with learning disorders and lower IQ as also noted in other studies (Lyytinen and Ahonen, 1988; Amiel-Tison et al., 1996; Vaivre-Douret et al., 2011a,b).

We showed that the MX subgroup comprises IM and VSC impairments in addition to comorbidities. The DCD children belonging to the MX group also exhibit disturbed motor planning and programing. Planning enables the project of a voluntary movement, firstly via an intention linked to the limbic and prefrontal cortex (often mental planning is intact verified by assessments of executive functions if not associated to PSR), and then by the organization of a motor plan for the sequences required, before executing the movement. It may also involve mental imagery. Motor planning requires correct integration of sensory information from the environment (tactile, visual, auditory) and from the body (kinaesthetic, proprioceptive, vestibular), for the movement produced to be suited to the situation. This is where disturbances may set in, for instance difficulty selecting the fingers in gestural imitation (concerning to IM impairment). The brain then specifies (programming) the parameters of the movement, that is to say the spatial-temporal aspects (direction, force to apply, amplitude, speed) and the visual-spatial elements (occipital-parietal dorsal pathway) that will orient action before the execution phase, which transits by basal ganglia, thalamus and cerebellum via the premotor area (Vaivre-Douret et al., 2011a,b, 2015).

We did not find any association between MRI abnormalities and PSR, DCD subtypes, or perinatal features in our samples. Thus, there is no evidence of neurological involvement or focal lesion in DCD children born full-term, but this could be attributable to the limitations of structural imaging. A recent interesting pilot study (Zwicker et al., 2012) using diffusion tensor shows that the axial diffusivity of the corticospinal tract and posterior thalamic radiation is lower and significantly correlated with the high degree of motor impairment in DCD children. This study appears to confirm our findings, associating PSR clinical investigations, while most studies (Foulder-Hughes and Cooke, 2003; Hadders-Algra et al., 2009) showed that MND or CP with DCD were linked to preterm birth or perinatal risk factors (intra-uterine growth retardation, low Apgar score).

Thus MND can be present with a mild form of pyramidal tract dysfunction, and go unnoticed. For instance phasic stretch reflex (PSR), leading to a discrete hemiparesia (distal) or mild spasticity in the lower limbs, can occur in the form of a co-morbidity which would be not considered in the DSM as an exclusive criterion attributable to a general medical condition coded on criterion D because there is no known neurological involvement, and the etiology is different compared to DCD. It is a mild developmental spasticity affecting distal muscles that are never examined in DCD studies. This is distinct from DCD etiology, but MND can coexist with DCD, with a high incidence (33%).

Our study underlines that DCD does not exclude a diagnosis of co-morbid mild form of CP without any neurological structural lesion. This is in contradiction with the hypothesis that DCD and CP have similar causal pathways and may lie on a continuum of movement disorders (Pearsall-Jones et al., 2010).

The nature of disorders in DCD subtypes defined by specific criteria suggests a dysfunction of the subcortical network (Lundy-Ekman et al., 1991; Ivry, 2003; Visser, 2003; Vaivre-Douret et al., 2011a, 2015; Lalanne et al., 2012) leading to disorders in the motor planning and/or programming of movement, in turn leading to disturbances in sensory-motor and spatial-temporal integration.

Evidence of MND suggests basal ganglia and cerebellum dysfunction: for instance synkinesia, dysdiadochokinesia, poor postural control, impaired of gesture quality, and/or timing or slower praxis. The MX subtype exhibits discriminant features in global motricity between upper and lower limbs and dexterity, with sensorimotor and perceptive-motor deficits which suggest subcortical and cortical disorders. It reflects a generalized dysfunction across the motor system and corpus callusum, possibly explaining more marked co-morbidity with learning disorders in the MX subtype. We found lower VIQ and PIQ scores in MX vs. VSC children, comparable to those in other studies (Lyytinen and Ahonen, 1988; Amiel-Tison et al., 1996; Visser, 2003; Vaivre-Douret et al., 2011a). It is interesting to note that Dazzan et al. (2006) found that smaller gray matter volume of cortical and subcortical structures of the sensori-motor system might be correlated with persistent MND in young healthy adults.

Furthermore, examination of laterality in the neurodevelopmental assessment, such as usual preference or dominant tonic laterality (see Table 1) is often not well-defined in the upper and lower limbs in the presence of marked PSR. Indeed, PSR raises tonicity by hypertonia on the affected side (hemiparesia). Since PSR in our sample was mainly present on the left, it could increase muscular tone on the left side of the body and disturb tone organization in the upper limbs. Thus a child with a usual laterality on the right could have a greater muscular tone in the left upper limbs than in the right, with consequences on motor coordination and motor control.

Our study has some limitations. Internal cross-validation was used for the multivariate statistical analysis to avoid overfitting the data, but the results need to be confirmed on an independent sample.

Furthermore, assessment of mild spasticity of dorsiflexion of the foot (PSR) requires clinical practice, but should systematically be undertaken even if Babinsky's reflex is normal. This investigation is nevertheless quick to implement.

In conclusion, the present study provides important new evidence in favor of implementing a complete neuropsychomotor physical assessment (with qualitative and quantitative measures), including neuromuscular tone examination, using appropriate standardized neurodevelopmental tools (common tasks across ages with normative data for age in order to distinguish motor command impairment (corticospinal tract) from motor planning or programming problems, all falling under the umbrella term of developmental coordination disorders. It enables better understanding of the nature of the neuropsychological and physiopathological causal pathways by taking account of possible co-morbidities, such as dysfunction of voluntary motricity linked to the corticospinal tract. This could contribute to improving diagnosis and defining suitable treatment programs in clinical practice, because MND can increase the expression of motor symptoms and their impact on outcome and prognosis in DCD children.

## Author contributions

LV conceived and designed the study, she contributed to collecting, analysing and interpreting of the data, contributed to drafting the article, reviewed and approved the final version and its submission. Her agreement covers all aspects of the work in ensuring that questions related to the accuracy or integrity of any part of the work are appropriately investigated and resolved. CL has substantially contributed to the design of the study, statistical analysis and interpretation of the data, and to drafting the article. He reviewed and approved the final version to be published and his agreement covers all aspects of the work. BG participated in the conception of the study and in the drafting of the article, approved the final version to be published and his agreement covers all aspects of the work.

### Conflict of interest statement

The authors declare that the research was conducted in the absence of any commercial or financial relationships that could be construed as a potential conflict of interest.

## Abbreviations

IMOG: Imitation of gestures
DIPR: Digital praxis
DIPE: Digital perception
MAND: Manual dexterity
CULL: Coordination between upper and lower limbs
BIDX: Bimanual dexterity (praxis)
RHYA: Rhythmic adaptation
OROP: Orofacial praxis
DYSD: Dysdiadochokinesia
AUDA: Auditory attention
DYNB: Dynamic balance
SYNK: Synkinesia
STAB: Static balance
HORP: Horizontal pursuit
HYPT: Hypotonia
PRSL: Praxis slowness
BSPI: Bodily spatial integration
EXEF: Executive functions
READ: Reading/spelling
ARTH: Arithmetic
WRKM: Working memory
VERP: Vertical pursuit
VISP: Visual perception
FISE: First sentences (language)
KINM: Kinaesthetic memory (perception)
KINM: Kinaesthetic memory
VISA: Visual spatial attention
SITA: Sitting alone
HYPK: Hyperkinesia
VISR: Visual refraction
VISC: Visual spatial construction
PUZL: Puzzles
AUDM: Auditory memory
VEPN: Visual evocated potentials
ORTH: Otorhinolaryngologia (Ear-Nose-Throat)
DRES: Dressing skills
POSC: Postural control
HAWR: Hand-writing
WALK: Walking alone
LEBL: Lego blocks
MOPA: Motor pathway
VISM: Visual spatial memory
DYGR: Dysgraphia
HMLS: Homogeneity of spontaneous manual laterality
CRAW: Crawling
HULU: Homogeneity of usual laterality upper/lower limbs
VISS: Visual spatial structuring
STDT: Sitting tone
HLUL, Homogeneity of tonic laterality upper/lower limbs.

## Glossary of Term:

DCD: Developmental Coordination Disorder
IM: Ideomotor DCD subtype
VSC: Visuo-spatial/visuo-constructional DCD subtype
MX: Mixed DCD subtype
MND: minor neurological dysfunctions
NSS: neurological soft signs
PSR: phasic stretch reflex
MRI: magnetic resonance imaging.
